# Biomedical Application Prospects of Gadolinium Oxide Nanoparticles for Regenerative Medicine

**DOI:** 10.3390/pharmaceutics16121627

**Published:** 2024-12-23

**Authors:** Ekaterina V. Silina, Natalia E. Manturova, Elena L. Chuvilina, Akhmedali A. Gasanov, Olga I. Andreeva, Maksim A. Pugachevskii, Aleksey V. Kochura, Alexey A. Kryukov, Yulia G. Suzdaltseva, Victor A. Stupin

**Affiliations:** 1 Institute of Digital Biodesign and Modeling of Living Systems, I.M. Sechenov First Moscow State Medical University (Sechenov University), 119991 Moscow, Russia; 2 Department of Hospital Surgery, Department of Plastic and Reconstructive Surgery, Cosmetology and Cell Technology, Pirogov Russian National Research Medical University (RNRMU), 117997 Moscow, Russiastvictor@bk.ru (V.A.S.); 3 “LANHIT” LLC, 105118 Moscow, Russia; chuvilina.elena@lanhit.ru (E.L.C.); akhmedali@lanhit.ru (A.A.G.); a.olga@lanhit.ru (O.I.A.); 4 Regional Nanotechnology Center, Southwest State University, 50 let Oktyabrya Str., 94, 305040 Kursk, Russia; maxpugachevskii@gmail.com (M.A.P.); akochura@mail.ru (A.V.K.); 5 Department of Pathophysiology, Kursk State Medical University, Karl Marx Str., 3, 305041 Kursk, Russia; krukovaa@kursksmu.net; 6 Vavilov Institute of General Genetics, Russian Academy of Sciences, Gubkin Str., 3, 119333 Moscow, Russia

**Keywords:** nanoparticles, gadolinium oxide, nanomaterials, nanogadolinium, regeneration, biomedicine, cytotoxicity, drug development

## Abstract

Background/Objectives: The aim was to study the possibilities of biomedical application of gadolinium oxide nanoparticles (Gd_2_O_3_ NPs) synthesized under industrial conditions, and evaluate their physicochemical properties, redox activity, biological activity, and safety using different human cell lines. Methods: The powder of Gd_2_O_3_ NPs was obtained by a process of thermal decomposition of gadolinium carbonate precipitated from nitrate solution, and was studied using transmission electron microscopy (TEM), X-ray diffraction (XRD), Raman spectroscopy, mass spectrometry, and scanning electron microscopy (SEM) with energy dispersive X-ray analyzer (EDX). The redox activity of different concentrations of Gd_2_O_3_ NPs was studied by the optical spectroscopy (OS) method in the photochemical degradation process of methylene blue dye upon irradiation with an optical source. Biological activity was studied on different human cell lines (keratinocytes, fibroblasts, mesenchymal stem cells (MSCs)) with evaluation of the effect of a wide range of Gd_2_O_3_ NP concentrations on metabolic and proliferative cellular activity (MTT test, direct cell counting, dead cell assessment, and visual assessment of cytoarchitectonics). The test of migration activity assessment on a model wound was performed on MSC culture. Results: According to TEM data, the size of the NPs was in the range of 2–43 nm, with an average of 20 nm. XRD analysis revealed that the f Gd_2_O_3_ nanoparticles had a cubic structure (C-form) of Gd_2_O_3_ ($Ia\bar{3)}$ with lattice parameter a = 10.79(9) Å. Raman spectroscopy showed that the f Gd_2_O_3_ nanoparticles had a high degree of crystallinity. By investigating the photooxidative degradation of methylene blue dye in the presence of f Gd_2_O_3_ NPs under red light irradiation, it was found that f Gd_2_O_3_ nanoparticles showed weak antioxidant activity, which depended on the particle content in the solution. At a concentration of 10^−3^ M, the highest antioxidant activity of f Gd_2_O_3_ nanoparticles was observed when the reaction rate constant of dye photodegradation decreased by 5.5% to 9.4 × 10^−3^ min^−1^. When the concentration of f Gd_2_O_3_ NPs in solution was increased to 10^−2^ M upon irradiation with a red light source, their antioxidant activity changed to pro-oxidant activity, accompanied by a 15% increase in the reaction rate of methylene blue degradation. Studies on cell lines showed a high level of safety and regenerative potential of Gd_2_O_3_ NPs, which stimulated fibroblast metabolism at a concentration of 10^−3^ M (27% enhancement), stimulated keratinocyte metabolism at concentrations of 10^−3^ M–10^−5^ M, and enhanced keratinocyte proliferation by an average of 35% at concentrations of 10^−4^ M. Furthermore, it accelerated the migration of MSCs, enhancing their proliferation, and promoting the healing of the model wound. Conclusions: The results of the study demonstrated the safety and regenerative potential of redox-active Gd_2_O_3_ NPs towards different cell lines. This may be the basis for further research to develop nanomaterials based on Gd_2_O_3_ NPs for skin wound healing and in regenerative medicine generally.

## 1. Introduction

Rapidly changing technologies in all areas of our lives are increasingly leading to the discovery and practical application of new knowledge. Over the past two decades, much attention has been paid to the specific effects of nanomaterials, which are often difficult to explain in terms of routine chemical, physical, and biological concepts. This explosive growth of interest is well illustrated by the increasing number of publications in the scientific literature, but while much has already been achieved in technical applications, we are only at the beginning of the road when it comes to medical aspects. The reason for this is the potential toxicity of metal nanoparticles [1,2] and the possibility of their unexpected penetration through cell membranes with unpredictable outcomes [3,4,5].

In 2006, the Medical Advisory Secretariat published a forecast on the development of nanotechnology in medicine and predicted that by 2024, nanotechnology solutions aimed at detection of DNA mutations and gene expression, creation of disease markers, drug delivery with controlled release, targeted tissue sequestration and thermal ablation of deep tumor tissues, multifunctional therapeutics with targeted drug delivery, and penetration enhancers will be introduced into clinical practice [6]. If we talk about practical medical applications today, of all the goals set by the Medical Advisory Secretariat, perhaps only gadolinium nanoparticles chelated to prevent toxic effects have become widely used as a contrast agent in magnetic resonance imaging (MRI) [7,8,9,10]. After the first such drug (Magnevist) appeared on the medical market in the 1980s, it was gadolinium (Gd) in the form of Gd_2_O_3_ chelates and, more often, Gd_2_ oxide that became widely used as a contrast agent for MRI, and today there are several tens of contrast agents based on Gd_2_O_3_ on the world market, due to its unique physical and chemical properties as well as high magnetic susceptibility of Gd^3+^ due to the presence of seven unpaired electrons (S = 7/2) at the 4f energy sublevel, which is extremely magnetic [11,12,13]. However, there are no other such successful examples in nanomedicine. This story well illustrates the difficulty of bringing any scientific project to practical application, especially in medicine, due to the super complexity of predicting the effects of nanoparticles or complexes including nanoparticles. However, judging by the number of publications in PubMed, the interest in nanotechnology in medicine has not only not weakened, but has increased manifold. According to PubMed, in 2003, just 890 sources were cited for nanoparticles, then in 2023, more than 33,000 articles on nanotechnology were published.

Of all known elements from the point of view of medical applications, the greatest attention has been paid to elements with variable valence, in particular the lanthanide group. Of this group, the largest number of studies has been devoted to cerium oxide nanoparticles, with 1362 publications in the past 5 years according to the PubMed database, including systematic reviews. Encouraging results indicating antimicrobial, regenerative, and antioxidant effects have been obtained, which makes nanoceria promising for drug development and applications in veterinary medicine and medicine [14,15,16,17]. However, the biomedical application of nanoceria in the form of final global products, unlike gadolinium, is still a prospect. Lanthanoid “gadolinium nanoparticles” showed with the same query a reference to only 615 publications for the past 5 years, and the absolute majority of works simply illustrated the high efficiency of gadolinium-based drugs in imaging (CT/MRI) examinations. According to PubMed data, only 67 studies have been devoted to gadolinium oxide nanoparticles in the past 5 years, and only a few of them are not related to contrasting, which determines the novelty of this field of knowledge. At the same time, taking into account that gadolinium is in the same lanthanide group as cerium, we can expect similar useful biological effects to those of nanoceria, which are also useful for regenerative medicine.

Gadolinium is a rare earth metal belonging to the lanthanide group (order number 64) with an atomic weight of 157.25 g/mol, and has 2 variants: -α-gadolinium with a hexagonal tightly packed crystal lattice with a = 3.6336 Å and c = 5.7810 Å at room temperature, and β-gadolinium with a cubic volume-centered crystal lattice with a = 4.06 Å at 1265 °C (2309 °F). In nature, the element occurs as a mixture of six stable isotopes. Odd-numbered isotopes have extremely high nuclear absorption cross sections, which explains its bright luminescence in radiological studies, and for gadolinium-157 it reaches 259,000 barns. As a result, the natural mixture of gadolinium isotopes also has a very high nuclear absorption cross section of 49,000 barns. For example, cerium has a nuclear absorption cross section 50,000 times smaller.

Gadolinium and its derivatives have been long and widely used as burn-up absorbers in the cores of nuclear reactors, in metallurgy to create alloys with special properties, as luminophores in medical visualization, and as a source of gamma radiation in electronic and optical devices. Medical (therapeutic) applications are much fewer. Nanoparticles containing gadolinium are used to deliver nanoparticles containing gadolinium to the primary tumor focus or its metastases for tumor control, delivery of antitumor drugs, and radiotherapeutic sessions of neutron-capture therapy [17,18,19,20,21,22,23,24] or phototherapy based on the same very high nuclear absorption cross section [25,26].

Activated controlled nanoparticles for X-ray irradiation with the inclusion of gadolinium have a double advantage: they imitate the effect of magnetic resonance imaging (MRI) contrast agent used in clinical practice and enhance the radiotherapeutic activity of conventional X-ray radiation [27]. This therapeutic effect is explained, on the one hand, by the paramagnetic properties of gadolinium and, on the other hand, by the formation of a high density of secondary radiation as a result of interaction between ionizing radiation and atoms with high atomic number, which leads to an increase in radiation dose in tumors where nanoparticles accumulate. The first results obtained in the treatment of breast cancer in patients with locally advanced cervical cancer and hepatocellular carcinoma are encouraging [28,29,30,31].

In addition to tumor diseases, gadolinium oxide nanoparticles have proven themselves in the diagnosis of ischemic and reperfusion myocardial lesions [32,33], in neurology [34,35], orthopedics [36], and hepatology [37]. The fluorescence effect of gadolinium nanoparticles under high-energy radiation has also been proposed for work with cell cultures as intracellular markers that allow observing the fate of each specific cell, which are well identified by MRI and fluorescence microscopy [38,39].

We had the goal of evaluating the potential biomedical applications of gadolinium oxide nanoparticles synthesized under industrial conditions. We believe that Gd_2_O_3_ nanoparticles hold great promise in the field of regeneration, including tissue engineering. In contrast to nanoceria, gadolinium oxide nanoparticles included in implants will be well visualized, which will make it possible to non-invasively study the evolution of the nanomaterial, which at the same time exhibits beneficial antioxidant properties. Confirmation of anti-apoptotic and antioxidant effects by Gd_2_O_3_ nanoparticles, which will be useful for accelerating wound healing, is reported in several studies [40,41,42,43,44]. The antioxidant effect of gadolinium nanoparticles was so obvious that there were even attempts to explain their success in the treatment of malignant tumors [45]. Complexes with nanogadolinium may also be useful in antimicrobial applications, which is especially relevant in view of the problem of antibiotic resistance and the wide spread of dangerous hospital infections [46,47,48,49,50,51,52]. In addition, the combinatorics of composition and methods for synthesizing of combinatorial compounds containing gadolinium oxide nanoparticles to improve biochemical and biological effects to accelerate wound healing, which are being conducted by many researchers and the results obtained by them, add more and more optimism to this research [53,54,55,56,57].

All of the above strengthened our intention to start a systematic study as members of a multidisciplinary research group including specialists in chemistry, physics, biology and medicine, and to evaluate the possibilities of using nanogadolinium in other biomedical applications, including those related to regenerative medicine.

The aim of the study was to synthesize nanosized gadolinium oxide powder, to evaluate its physicochemical characteristics, to investigate its pro- and antioxidant properties, and to study its effect on cytotoxicity, metabolism, and proliferative and migratory activity of different cell lines involved in regeneration (multipotent stromal cells, fibroblasts, keratinocytes).

## 2. Materials and Methods

### 2.1. Synthesis of Gadolinium Oxide Nanoparticles

Nanosized gadolinium oxide was obtained in the production conditions of the research and production complex of LLC “LANHIT” by a typical process of thermal decomposition of gadolinium carbonate precipitated from nitrate solution. The processes are described by Equations (1)–(3):

Gd_2_O_3_+ 6HNO_3_→2Gd(NO_3_)_3_ +3H_2_O (1)

2Gd(NO_3_)_3_ + 3(NH_4_)_2_CO_3_→Gd_2_(CO_3_)_3_↓ + 6NH_4_NO_3_ (2)

Gd_2_(CO_3_)_3_→Gd_2_O_3_ + 3CO_2_↑ (3)

As initial reagents, we used gadolinium oxide Gd_2_O_3_ with 99.99% purity in terms of metal impurities (metals basis) (LANHIT, Moscow, Russia), extra pure nitric acid, chemically pure ammonium carbonate (NH_4_)_2_CO_3_, and ammonium nitrate NH_4_NO_3_. The precipitation was performed from gadolinium nitrate solution with a concentration of 50 g/L gadolinium oxide. A solution of ammonium carbonate (NH_4_)_2_CO_3_ with a concentration of 200 g/L was used as a precipitant. Into a reaction vessel with volume of 3 L 1 was poured 1 L of freshly prepared ammonium nitrate solution (as a crystallization regulator) with concentration of 4 mol/L, then from one separating funnel was added dropwise 1 L of gadolinium nitrate solution with a concentration of 50 g/L, and from another—500 mL of ammonium carbonate solution with a concentration 200 g/L. The solutions of gadolinium nitrate and precipitant were delivered equally and simultaneously into the reaction vessel under continuous stirring with a Hei-TORQUE stirrer (Heidolph Instruments, Schwabach, Germany) at 300–350 rpm for 10 min.

After complete delivery of reagents, the obtained gadolinium carbonate was filtered in a Buechner funnel and washed with distilled water through a filter. The temperature of the wash water was 30–40 °C and the volume was 2 L. Then, the precipitate was dried in air at 25–35 °C for 2–3 days to obtain a bulk product.

Decomposition of carbonate was carried out in a quartz crucible placed in a muffle furnace at 650 °C for 3 h. After cooling, the obtained gadolinium oxide was sieved through a polyamide fiber mesh with a mesh size of 0.1 mm.

Powders and aqueous suspensions (solvent—water for injection) of Gd_2_O_3_ NPs in a wide range of concentrations—from 3.6 g/L (10^−2^ M) to 0.0005 g/L (10^−6^ M) taking into account the molecular weight of Gd_2_O_3_ 362.5 g/mol—were prepared for physicochemical and biological methods of investigation.

For biological studies, each concentration of nanogadolinium was prepared in an individual tube. For this purpose, powdered Gd_2_O_3_ NPs with sterile water for injection were stirred in a magnetic stirrer for 30 min, after which they were dispersed in an ultrasonic bath for 20 min, and then centrifuged at 4000 rpm for 10 min to select the smallest fraction of nanoparticles. Next, the concentration in the final suspension was determined by thermogravimetry, after which 4 final concentrations of Gd_2_O_3_ NPs were created for cell culture studies: 10^−2^ M, 10^−3^ M, 10^−4^ M, 10^−5^ M.

### 2.2. Physicochemical Methods of Research

The synthesized powder of Gd_2_O_3_ NPs was analyzed by mass spectrometry, transmission electron microscopy (TEM), X-ray diffraction (XRD), Raman spectroscopy, and scanning electron microscopy (SEM) with energy dispersive X-ray analyzer (EDX). Liquid forms of nanogadolinium (suspensions of different concentrations) were investigated for redox activity using the optical spectroscopy (OS) method in the photochemical degradation process of methylene blue dye upon irradiation with an optical source.

The size of Gd_2_O_3_ particles was determined by transmission electron microscopy (TEM) on a JEM2100 device at an accelerating voltage of 200 kV (JEOL, Akishima, Japan). Overview images were obtained at magnifications (×10,000–20,000). For more detailed study of the structure and observation of traces of atomic planes, we used direct magnification ×400,000—up to 1.5 million times, the maximum approximate ruler on microphotographs—10 nm. Selector apertures were used to select areas and obtain diffraction patterns.

Image J 1.54 and Statistica 10.0 programs were used to estimate the average size of nanoparticles. The diameters of 200 nanograins in different grid regions were measured, after which the variance was divided into equal size intervals. Finally, a frequency histogram was constructed and the average size of nanoparticles was determined.

The X-ray diffractogram (XRD) of the Gd_2_O_3_ NPs powder sample was measured at room temperature using a GBC EMMA X-ray diffractometer (Scientific Equipment, Victoria, AU, USA) at wavelength λ = 1.5401 Å of the CuKα line in θ–2θ geometry with 0.02° step. The obtained data were processed using the Rietveld method with the PowderCell program [58]. The theoretical diffractogram was calculated from the structural parameters of the Gd_2_O_3_ lattice determined in [59] from synchrotron radiation diffraction experiments.

The vibrational Raman spectroscopy was performed on a confocal Omega Scope TM Raman spectrometer (AIST-NT Inc., Edison, NJ, USA). Excitation was performed with a laser with a wavelength of 532 nm, with a power of 30 mW in the region of 150–1000 cm^−1^. A diffraction grating with a density of 1800 mm^−1^, providing a spectral resolution in the measured region of 0.8 cm^−1^, was used to obtain the spectrum of excited radiation.

The impurity composition of the initial and obtained oxides was analyzed by spark source mass spectrometry with a dual focus JMS-01-BM2 (JEOL, Akishima, Japan). The high-resolution mass spectra were photographed on Ilfrod-Q plates. A Joyce Loebl (UK) MDM6 microdensitometer and a NOVA 4 (USA) online mini-computer were used for the mass spectrum lines’ identification. Quantification was calculated using GC- MS Lab software. Noble gases and transuranic elements have not been tabulated because their concentrations were lower than detection limits (<0.01 ppm). The impurity content was represented as parts per million, relative to the metal matrix (1 ppm = 0.0001%). The mass of powder sent for the study was 25 g.

Surface morphology and structure features, as well as the distribution and quantitative composition of chemical elements in the studied samples were investigated using a scanning electron microscope (SEM) JSM-6610LV (JEOL Ltd., Akishima, Japan) in high-vacuum mode. The spatial resolution of the microscope in high vacuum mode was 3 nm. The accelerating voltage was 20 kV. For elemental analysis, the studies were carried out using a characteristic X-ray detector (XRMS/EDS—X-ray spectral microanalysis), which allows studying the surface of a sample’s microspectral distributions of chemical elements in the range from Be (Beryllium) to Pu (Plutonium). The active area of the silicon window of the X-Max Silicon Drift Detector, Oxford Instruments, Great Britain, was 20 mm^2^. The stability of the detector resolution and the stability of the peak positions in the spectrum were not less than ±1 eV.

The antioxidant and prooxidant properties of Gd_2_O_3_ NPs in a wide range of concentrations were investigated using an HR-2000 optical spectrometer (Ocean OpticsInc, Dunedin, FL, USA) during a photooxidative degradation reaction of methylene blue dye (Himedia Laboratories Pvt. Ltd., Maharashtra, India), which was both a photosensitizer (with absorption maximum at the wavelength of 662 nm) and an indicator of the process. During irradiation of methylene blue by a light source with a wavelength of 660 nm due to strong absorption of optical photons by the dye, there was photogeneration of electron-hole pairs initiating a cascade of oxidation-reduction reactions in solution with the formation of active radicals. In this case, degradation of methylene blue and highlighting of the solution based on it occurred due to the formation of highly active oxygen radicals, in particular short-lived hydroxyl radicals, which attacked methylene blue molecules and destroyed its coloring centers.

Irradiation of methylene blue solutions was carried out using a red light-emitting diode source in a narrow spectral range with a wavelength of 660 ± 5 nm. The irradiation dose was 80 J/cm^2^. Kinetic dependences of the photodegradation process in all cases corresponded to the pseudo-first order reaction. The activity of photodegradation processes was characterized by the reaction rate constant according to Formula (4):

(4) $$k=-\ln(\frac{C}{C_{0}})/t$$

When Gd_2_O_3_ NPs were added, their antioxidant or pro-oxidant properties were manifested, leading to either a slowing down or acceleration of the reaction rate of dye degradation, respectively.

### 2.3. Biomedical Methods of Research

The study was performed using different cell lines involved in wound healing (on cell cultures of human keratinocytes, human fibroblasts, and human mesenchymal stem cells) to determine the regenerative potential of Gd_2_O_3_ NPs.

Since the final goal of the study was to obtain a prototype of a medical product, we tried to conduct toxicological studies as thoroughly as possible in a wide range of upper limit concentrations. The concentrations of NPs presented in the study (10^−2^ M, 10^−3^ M, 10^−4^ M, 10^−5^ M) are individual chemical samples that were added to culture wells in a volume of 10% (10 vol.%) Therefore, the actual concentration in the cell culture was 10 times less (10^−3^ M–10^−6^ M). For example, we added 100 µL of NPs at a concentration of 10^−3^ M to 900 µL of medium + cells, so the final concentration Gd_2_O_3_ NPs in the well was 10^−4^ M, while in tables and graphs this example is represented by a group of 10^−3^ M (analogy with the concentration of substances in medicines).

First, Gd_2_O_3_ NPs were tested to evaluate the effects of different concentrations (10^−2^ to 10^−6^ M) on the metabolic and proliferative activities of human fibroblasts (MTT test, direct cell counting after 72 h of co-culture). Visualization of cells under light microscopy showed unaffected agglomeration of nanoparticles at high concentration and difficulty in performing cell counting and their focusing. Therefore, due to the agglomeration of NPs at a concentration of 10^−2^ M and the impossibility of performing an accurate cell count, further culture studies (MTT test, direct cell counting with visualization and cell viability assessment) were performed on human keratinocyte culture after 72 h of co-culture with nanoparticles at concentrations of 10^−3^ M, 10^−4^ M, and 10^−5^ M.

Based on the obtained data from the above series of experiments on fibroblasts and keratinocytes, the best (promising) concentration of Gd_2_O_3_ NPs (10^−4^ M) was selected and a test was performed to evaluate migration activity on a culture of human mesenchymal stem cells on a model wound.

#### 2.3.1. Methods for Evaluating the Effect of Different Concentrations of Aqueous Suspensions of Gd_2_O_3_ NPs, on Cytotoxicity/Biocompatibility, Metabolic, and Proliferative Activity of Human Fibroblasts and Keratinocytes

##### Cell Lines of Fibroblasts and Keratinocytes and Their Cultivation

Human fibroblast cell line BJ TERT was sourced from American Type Culture Collection ATCC (Manassas, VA, USA). The cells belong to the hTERT-immortalized type obtained by transfection of the BJ fibroblast cell line with a plasmid-expressing telomerase reverse transcriptase (hTERT).

Human keratinocytes from the HaCaT cell line, which are spontaneously immortalized non-cancerous human keratinocytes, are capable of unrestricted division while exhibiting normal differentiation [60,61]. Cell source: adult human skin. Line origin: FSBI N.N. Blokhin National Medical Research Center for Oncology of the Ministry of Health of the Russian Federation (Moscow, Russia).

Human fibroblasts of the BJ TERT line were cultured in commercially treated Petri dishes, d 100 mm (SPL Life Sciences, Pocheon, Republic of Korea), in DMEM medium containing at least 4.5 g/L glucose (PanEco, Moscow, Russia) supplemented with 10% fetal calf serum (Global Kang Biotechnology, Building, China), 146 mg glutamine (PanEco, Moscow, Russia), and 1% penicillin, plus 1% streptomycin (PanEco, Moscow, Russia). Cells were incubated at 5% CO_2_ concentration in ambient air at 37 °C and controlled humidity under standard CO_2_-incubator conditions (Binder, Baddeckenstedt, Germany). When the fibroblasts reached 100% confluency, the next passage of cell culture was performed; the medium was changed after three days from the moment of passage. Cultivation of human fibroblasts of the BJ TERT line was performed according to the standard protocol.

Then, according to the design of the experiment, human fibroblasts were seeded into 24-well plates (SPL Life Sciences, Pocheon, Republic of Korea) at a cell concentration in suspension of 5.0 × 10^4^/mL, and counting was performed in a standardized method using Countess II Automated Cell Counter (Thermo Scientific, Waltham, MA, USA). Cells were placed into wells with a volume of 900 μL. After 24 h of cultivation under standard CO_2_ incubator conditions, 100 μL per well was added to the fibroblasts at the appropriate concentrations, and the coincubation was continued under the standard conditions described above for the next 72 h.

Cells were cultured in Petri dishes (SPL LifeSciences, Pocheon, Republic of Korea) in DMEM medium with high glucose content (at least 4.5 g/L) (PanEco, Moscow, Russia), supplemented with 10% fetal calf serum (Global Kang Biotechnology, Building, China), 146 mg glutamine (PanEco, Moscow, Russia), and 1% penicillin, plus 1% streptomycin (PanEco, Moscow, Russia). Cell cultures were incubated in a CO_2_ incubator (Binder, Germany) under standard controlled conditions (5%CO_2_, 37 °C) and controlled humidity. Passage of HaCaT keratinocytes was performed every 3 days according to the standard protocol. For the experiment, cells were seeded in 24-well plates (SPL LifeSciences, Republic of Korea) using DMEM medium according to the standard protocol at a concentration of cells in suspension of 7.0 × 10^4^/mL. After 24 h, the test substances (nanogadolinium of different concentrations—10 vol%) were added and co-incubation was continued for the next 72 h. The initial solvent, which was sterile water for injections, was added as a control.

##### MTT-Test

To assess the metabolic activity of cells, a colorimetric MTT-test was performed, during which yellow tetrazole was reduced in living cells into purple formazan. The amount of formazan formed as a result of this reaction was proportional to the number of viable cells in the well.

After 72 h of co-incubation with nanosubstances, the culture medium was removed from the wells and 3% MTT reagent (PanEco, Moscow, Russia) was added. To realize the intracellular reaction, the plate with the reagent was placed at 37 °C for 30 min. The MTT working solution was then removed and dimethyl sulfoxide (DMSO) was added as a solubilizing agent. The plate was incubated at room temperature on an oscillating shaker (Elmi-S4, Riga, Latvia) for 5 min, then the dissolved contents were transferred in a volume of 100 µL into a 96-well plate to measure the optical density at 540 nm spectrophotometrically (Multiscan Labsystems, Vantaa, Finland). The final result was expressed in relative optical density (OD) units.

##### Determination of Proliferative Activity and Dead/Living Cells Ratio with Trypan Blue Staining

Cell counting was performed automatically using a Countess II Automated Cell Counter (Thermo Scientific, Waltham, MA, USA), which reflected the quantitative composition of the cell suspension and made it possible to identify viable cells in the population. This method consisted in visualizing and recognizing cells after they were stained with special dyes that penetrated the cytoplasm when the cell wall was damaged. At the end of the incubation period, the medium was removed from the plate and the cell wells were washed with phosphate–salt buffer. Next, cells were detached by adding trypsin-Versen solution in a 1:3 ratio (PanEco, Moscow, Russia) for 1 min at 37 °C. Then, 10 µL of trypan blue dye (PanEco, Moscow, Russia) was added to the cell suspension and mixed by pipetting. The stained cell suspension was added to the slide for automatic counting and fixation of cell viability. As a result, the total concentration of cells in 1 mL and the percentage of live and dead cells were counted.

#### 2.3.2. Study of Migration Activity and Rate of Healing of a Model Wound In Vitro on Human Mesenchymal Stromal Cell Culture

##### Culture of Human Adipose-Derived Mesenchymal Stromal Cells

Human mesenchymal stromal cells (MSC) lines were used in this study. MSCs were previously generated in our laboratory using the method described in [62]. Cells at passage 3 and 4 were used for experiments.

MSCs were cultured in a humidified atmosphere containing 5% CO_2_ at 37 °C on standard plastic dishes for cell culture (Corning Costar, Corning, NY, USA) in Dulbecco’s Modified Eagle Medium/Nutrient Mixture F-12 (DMEM/F12; Thermo Fisher Scientific, Waltham, MA, USA) supplemented with 100 U/mL penicillin and streptomycin, 2 mM L-glutamine (Paneco, Moscow, Russia), and 10% fetal bovine serum (One Shot FBS; Gibco, Thermo Fisher Scientific, Waltham, MA, USA) until ~70–80% confluence. The medium was changed every 3–4 d. Then MSCs were passaged with trypsin/EDTA solution (Paneco, Moscow, Russia). The identity of expanded MSCs was confirmed by the minimal criteria proposed by The International Society for Cellular Therapy [63].

MSCs from passage 3 attached to the plastic surface were positive for CD73, CD90, and CD105 expression, and negative for CD34, CD45, and CD14 expression. These cells were able to differentiate into adipogenic, osteogenic, and chondrogenic lineages under special conditions as described in [62].

##### Methodology for Assessment of Migration Activity and Rate of In Vitro Model Wound Healing on Human MSCs

The migration rate of the MSCs was assessed upon the addition of Gd_2_O_3_ NPs at concentrations of 10^−4^ M. The cells in the medium alone were used as a control. The scratch size was initially set to be 100%, and wideness was measured at different time points—immediately after adding NPs (point 0), after 24 h, 48 h, and 72 h.

MSCs were grown under the same conditions as those described in the previous section. The cell layer was then scratched across the diameter of 35 mm Petri dishes using 200 μL pipette tips (Greiner Bio-One, Kremsmunster, Austria). The culture medium was then immediately removed (along with any dislodged cells) and replaced with 1.5 mL of DMEM/F-12 containing 10% FBS, with the addition of 10^−4^ M Gd_2_O_3_. Images from the same viewpoint were obtained under microscopy at the baseline (0 h) and every 24 h after wounding for determination of the extent of wound closure. ImageJ 1.49v (National Institutes of Health, Bethesda, MD, USA) was used to measure the void area, and the extent of the wound closure was evaluated (width (%) = void area/baseline area) in each group.

At each control point (0, 24 h, 48 h, 72 h), four measurements were performed by two independent people using the same method, thus obtaining 12 quantitative variables for each group at each time point.

### 2.4. Statistical Data Processing

Statistical processing of the results of the study on cell lines was performed using the statistical program SPSS 25.0 (IBM Corp., Armonk, NY, USA).

First of all, the normality of the distributions of MTT-test and cell count indices for each of the samples was assessed by the Kolmogorov–Smirnov and Shapiro–Wilk criteria. The normality test proved the obedience to the law of normal distribution (*p* > 0.05).

Descriptive statistics of continuous quantitative measures are presented as mean, std. deviation, std. error, 95% confidence interval for mean (95CI), minimum, and maximum. The *t*-test was used to compare only two groups. One-factor ANOVA analysis of variance was performed for comparative analysis of the different subgroups. A posteriori multiple comparisons were performed using the Dunnett test (for comparison with the control). Differences were considered statistically significant when the *p*-value was < 0.05.

## 3. Results

### 3.1. Results of Evaluation of Physicochemical Characteristics of Gadolinium Oxide Nanoparticle Powder

#### 3.1.1. Transmission Electron Microscopy

**Figure 1:**
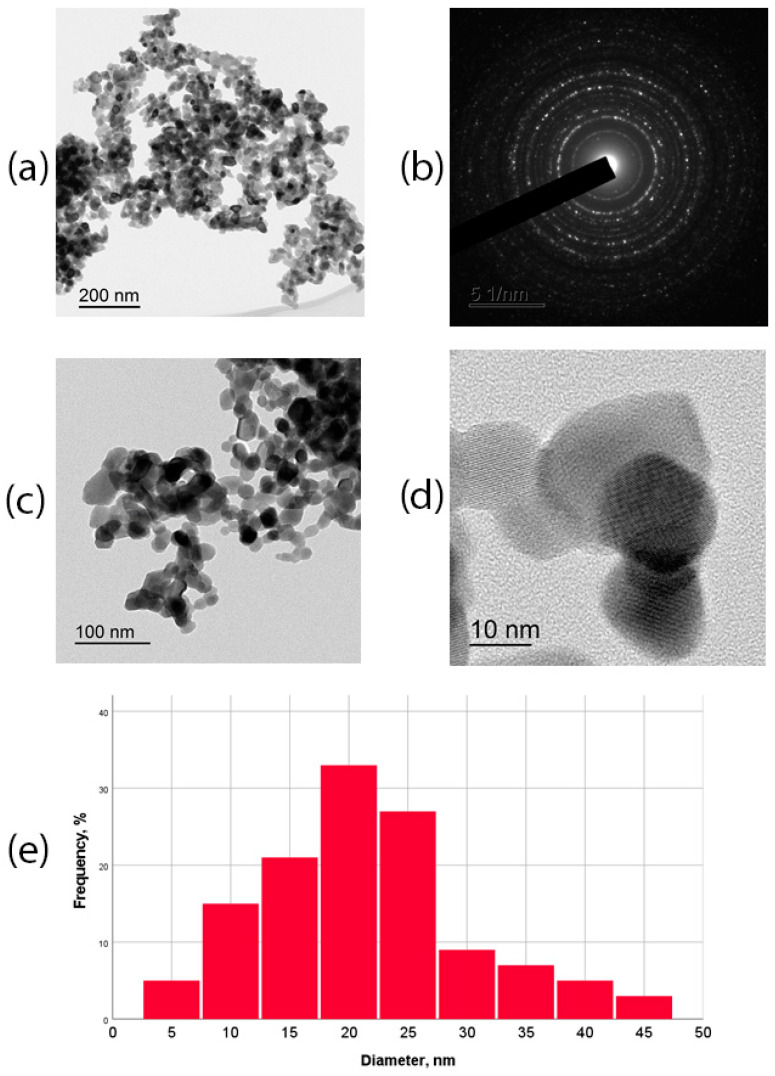
TEM images of powdered Gd_2_O_3_ NPs, obtained on a JEM-2100 microscope at accelerating voltage 200 kV: (**a**) overview image of agglomerate and (**b**) its electronogram; (**c**,**d**) enlarged image before visualization of separate nanoparticles with scale bar 100 nm–10 nm; (**e**) size distribution of Gd_2_O_3_ NPs.

The study of Gd_2_O_3_ by transmission electron microscopy (Figure 1) showed that the shape of the particles was close to cubic. The size of the nanoparticles ranged from 2 nm to 43 nm, with the maximum of the distribution corresponding to 15–25 nm. Gd_2_O_3_ particles with a diameter ≤ 10 nm were 18%. The average size of the nanoparticles was 23 ± 9 nm.

#### 3.1.2. X-Ray Diffraction

**Figure 2:**
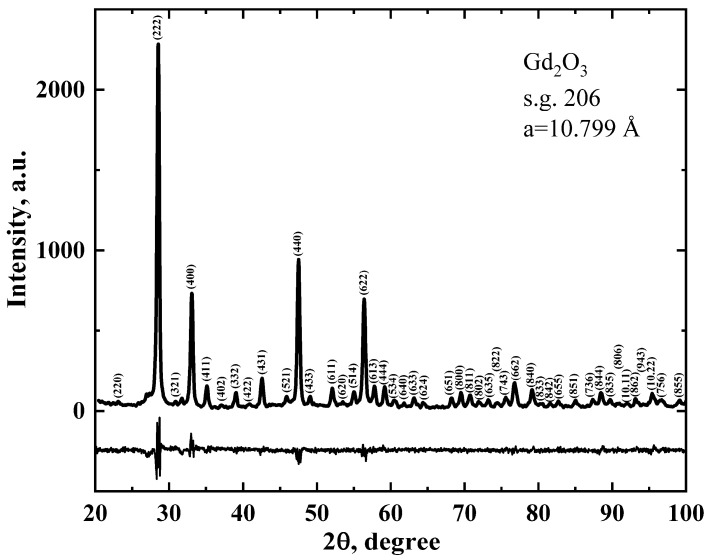
Diffractogram of a sample of Gd_2_O_3_ powder. The upper graph represents the experimental curve with indexing of peaks by the corresponding planes from which the diffraction occurred. The lower graph represents the difference between the experimental and theoretical diffractograms.

X-ray phase analysis (XRD) showed (Figure 2) that almost all diffraction peaks, both in position and intensity, corresponded to the cubic structure (C-form) of Gd_2_O_3_ ($Ia\bar{3}$, p.g. 206) with the lattice parameter a = 10.79(9) Å, which was very close to the values obtained earlier for the nanoscale powder Gd_2_O_3_: 10.8175 Å [59], 10.813 Å [64]. However, a small number of weak diffractions were present in the diffractogram, which could indicate the presence of defects in the crystal structure and the manifestation of forbidden diffractions.

The coherent X-ray scattering length D was determined by the Debye–Scherrer formula: D = Kλ/(βcosθ), where θ is the Bragg angle, λ is the X-ray wavelength, β is the half-width (width at half-height) of the diffraction peak, and K is a coefficient depending on the particle shape, which can vary from 0.6 to 2.1. If the particle shape is close to cubic, K depends on the moduli of the indices of the plane (*hkl*) from which the diffraction occurred: K = 6h^3^/[(h^2^ + k^2^ + l^2^)^1/2^(6h^2^ − 2(k + l)h + kl] [65]. The value of crystallite size in the studied sample of Gd_2_O_3_ determined by diffractions with the highest intensity was 23 ± 3 nm.

#### 3.1.3. Raman Spectroscopy

**Figure 3:**
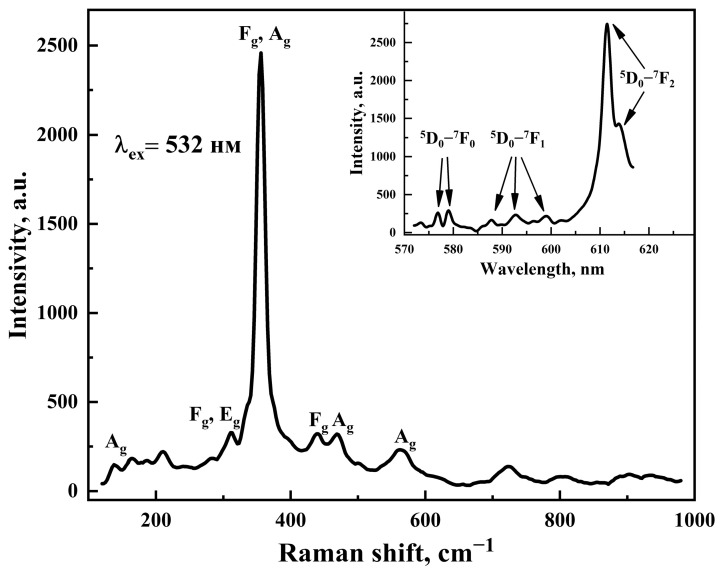
Raman spectrum of Gd_2_O_3_ powder with identification of the main Raman active modes. Inset photoluminescence spectrum of the sample near the wavelength of excitation radiation with marked energy transitions due to the presence of Eu^3+^ ions in the crystal lattice of Gd_2_O_3_.

Raman spectra of Gd_2_O_3_ powder are shown in Figure 3. According to the group theoretic analysis $Ia\bar{3}$, there are 22 allowed Raman vibrations for the cubic structure—these are modes 4Ag, 4E_g_ and 14 F_g_ [66]. Due to the large difference in the weight of Gd and O ions, the Raman spectrum of Gd_2_O_3_ is divided into three regions: (1) at frequencies less than 250 cm^−1^, the vibrations of heavy gadolinium ions predominate; (2) in the range from 250 cm^−1^ to 500 cm^−1^, the vibrations across the bends (bending vibrations) of Gd–O are the main ones; (3) in the region 550–1000 cm^−1^, the vibrations along the bends (stretching vibrations) of Gd–O may be observed. Since the allowed Raman modes are quite numerous, oscillations corresponding to the combination of phonon modes (with total or difference frequencies) should be observed in all the abovementioned regions of the spectrum. The region above 1000 cm^−1^ was not investigated by us because it does not characterize the lattice vibrations, but it can be used in the analysis of the luminescence response from impurity centers [67]. The measured Raman spectrum (Figure 2) was consistent with previously observed Raman spectra for single crystals, films, and nanoparticles of Gd_2_O_3_ [68,69]. The obtained spectrum contained peaks in all three regions described above, and their position was in good agreement with the position of Raman active modes (the strongest of which are marked in Figure 3), and with the position of modes caused by the main vibrations of atoms in the crystal lattice (Gd–Gd and Gd–O) or their combinations, which spoke about the structural quality of Gd_2_O_3_ nanocrystals.

According to the parameters of the strongest Raman mode, we can estimate the size of Gd_2_O_3_ nanoparticles using the correlation from [70]: FWHM (cm^−1^) = 10 + 124.7/D (nm), where FWHM is the half-width of the main Raman mode and D is the crystallite size. For the mode 356 cm^−1^, FWHM = 15.9 cm^−1^ and, respectively, the average size of the Gd_2_O_3_D ≈ 21 nm, which was almost in agreement with the values determined from TEM experiments and XRD analysis.

As shown by detailed analysis of the Raman spectrum, in Gd_2_O_3_ the spectral region ω > 1000 cm^−1^ did not characterize the lattice vibrations, but was used in the analysis of the luminescence response from impurity centers [67], since the photoluminescence efficiency of rare-earth metal impurities in their oxides can be quite high [71,72,73,74] due to the effect of energy transfer from the cation to the impurity ion. The inset to Figure 3 shows a part of the Raman spectrum containing photoluminescent peaks. They are well identified with the energy transitions involving impurity ions Eu^3+^: the transitions ^5^D_0_–^7^F_1_ and ^5^D_0_–^7^F_2_ are formed due to magnetic and electric dipole interactions, respectively, and the transitions ^5^D_0_–^7^F_0_ for ideal crystals are forbidden by the selection rules, but in real crystals and, even more so, nanoparticles, due to distortions of the crystal field these transitions can be observed [74].

#### 3.1.4. Scanning Electron Microscopy and Energy Dispersive Analysis of Secondary (Characteristic) X-Rays

**Figure 4:**
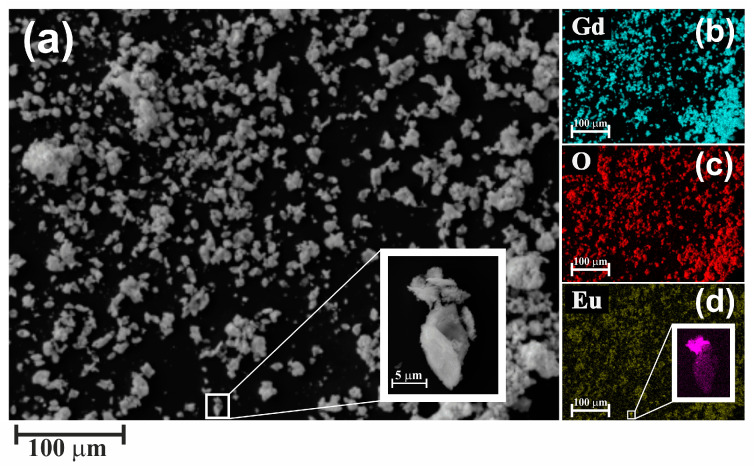
Images of Gd_2_O_3_ powder particles obtained by scanning electron microscopy (**a**) and elemental distribution maps: Gd (**b**), O (**c**), and Eu (**d**). The insets show the image of the agglomerate with increased Eu content.

SEM images (Figure 4) show that the powder consisted mainly of agglomerates. The size of the constituent Gd_2_O_3_ nanoparticles could be evaluated only at high magnifications; on average, the size of the nanoparticles was not more than 30 nm. The average size of agglomerates was 30 μm, the maximum size was 60 μm, and the minimum size was 10 nm.

Energy dispersive analysis of secondary X-rays was used to determine the elemental composition. The composition of the powder was within the measurement error (0.2% at.) equal to the stoichiometric composition (Figure 4b,c) when scanning on small scales (multiplicity 500–100), and the presence of impurity was detected only with multiple scans of the investigated area. In the overwhelming part of agglomerates its concentration was much less than 0.2% at. and could not be accurately estimated by the used methodology. However, when plotting the europium distribution map, single agglomerates containing europium in increased amounts were clearly visible (Figure 4a,d insets). On average for such particles, the europium concentration reached 4.5% at. while maintaining the stoichiometry of the initial compound.

#### 3.1.5. Mass Spectrometry

The study of purity of synthesized gadolinium oxide nano powder by mass spectrometry data showed that the product contained the following main impurities: rare-earth metals—Ho (20 ppm), La (6 ppm), Eu and Tb (3 ppm), Tm (2 ppm), Sm (1 ppm), with others (up to 1 ppm, all rare-earth metals) less than 15 ppm (Table 1). The final purity of the product Gd_2_O_3_ was 99.9%.

### 3.2. Result of Evaluation of the Redox Activity of Gd_2_O_3_ Nps at Different Concentrations

**Figure 5:**
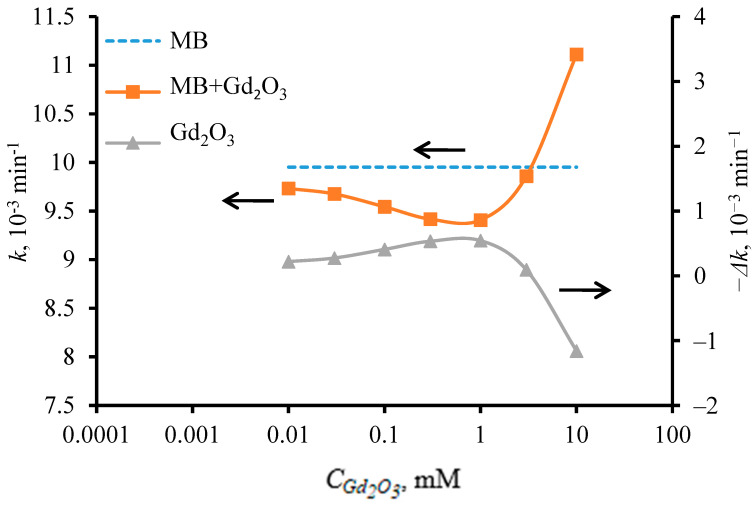
Dependence of the photodegradation rate constant of methylene blue and its variation on the concentration of Gd_2_O_3_ NPs under red light irradiation. Dashed line shows (MB) methylene blue without addition of Gd_2_O_3_ NPs. The arrows indicate that both the blue dashed line (MB) and the orange solid line (MB+Gd_2_O_3_) belong to the left axis (*k)*, and the gray solid line (Gd_2_O_3_) belongs to the right axis (*–Δk).*

Figure 5 shows the results of the redox activity of Gd2O3NPs in the photooxidative degradation process of methylene blue dye under red light irradiation. The change in the reaction rate was determined and calculated by the formula ${∆k}_{{Gd_{2}O}_{3}}=k_{MB+{Gd_{2}O}_{3}}-k_{MB}$. The minus sign on the auxiliary Y axis of the figure indicates that the degradation reaction of methylene blue was slowed down in the presence of Gd_2_O_3_ nanoparticles with a concentration ≤ 10^−3^ M. The figure shows that Gd_2_O_3_ NPs generally had a weakly pronounced antioxidant activity, which depended on the concentration of particles in solution. Thus, the highest antioxidant activity was observed for particles with a concentration of 10^−3^ M, when the photodegradation rate constant of methylene blue decreased by 5.5% to 9.4∙10^−3^ min^−1^. When the particle content was reduced to 10^−5^ M, the antioxidant activity decreased to 2.2%, due to the decrease in the amount of active ingredient. Interestingly, as the concentration increased above 10^−3^ M, a decrease in antioxidant activity was also observed, and at a concentration of 10^−2^ M, the activity of Gd_2_O_3_ NPs became pro-oxidant, accelerating the reaction rate of methylene blue degradation by 15%.

It is noted in [75,76] that lanthanide oxides may exhibit dose-dependent antioxidant activity due to changes in the degree of oxidation of cations on the oxide surface. This may explain the manifestation of weak antioxidant properties of Gd_2_O_3_ NPs at concentrations of 10^−3^ M and below. However, at high particle concentration of 10^−2^ M, the sign of chemical activity was reversed, and the particle activity became pro-oxidant, which was possibly due to a competing process. For Gd_2_O_3_ NPs, this process can be related to the presence of impurity elements (Ho, Eu, Yb) in the crystal matrix of gadolinium oxide, which generate additional energy levels inside the forbidden band of gadolinium oxide.

### 3.3. Results of Biological Studies on Cell Lines

#### 3.3.1. Results of Studies of the Effect of Different Concentrations of Aqueous Suspensions of Gd_2_O_3_ NPs on Cytotoxicity, Metabolic, and Proliferative Activity of Human Fibroblasts

**Figure 6:**
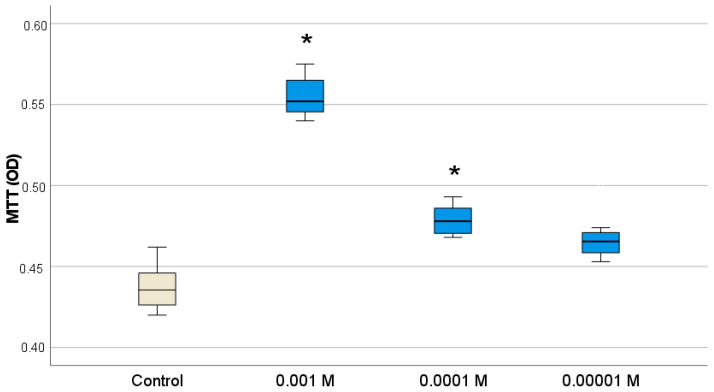
Effect of different concentrations of Gd_2_O_3_ NPs on metabolic activity of human fibroblasts in MTT test (* difference from control at *p* < 0.001, Dunnett post hoc tests).

**Figure 7:**
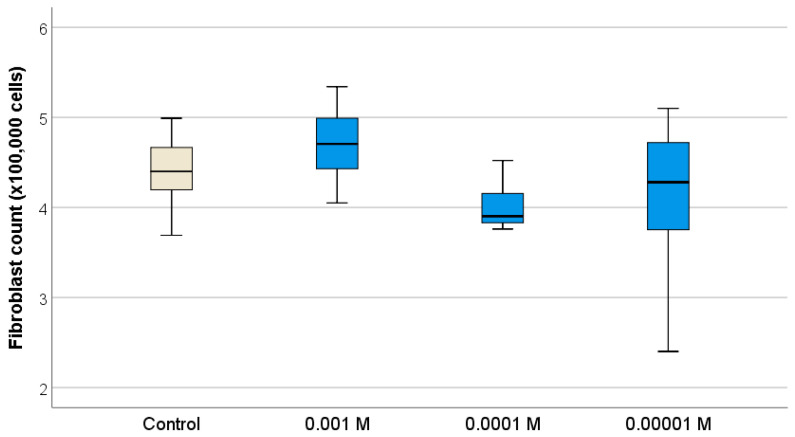
Effect of different concentrations of Gd_2_O_3_ NPs on proliferative activity of fibroblasts by direct cell counting using an automated cell counter (*p* = 0.142).

According to the MTT test, we found that Gd_2_O_3_ NPs sols significantly activated the metabolism of human fibroblasts in a wide range of concentrations (10^−2^–10^−4^ M) (Figure 6), without affecting their proliferation at all concentrations that we studied (Figure 7). Interestingly, the greatest increase in OD was recorded when co-cultured with the highest concentrations of nanogadolinium (the increase in metabolic activity was on average 1.27-fold at concentration 10^−3^ M relative to control wells (*p* < 0.0001). When the concentration of Gd_2_O_3_ NPs was reduced to 10^−4^ M and 10^−5^ M, the OD index exceeded the control on average by 1.10 and 1.07 times, respectively, but the proliferative activity at low concentrations was characterized by an unreliable tendency to decrease by 7–10% (Table 2).

**Figure 8:**
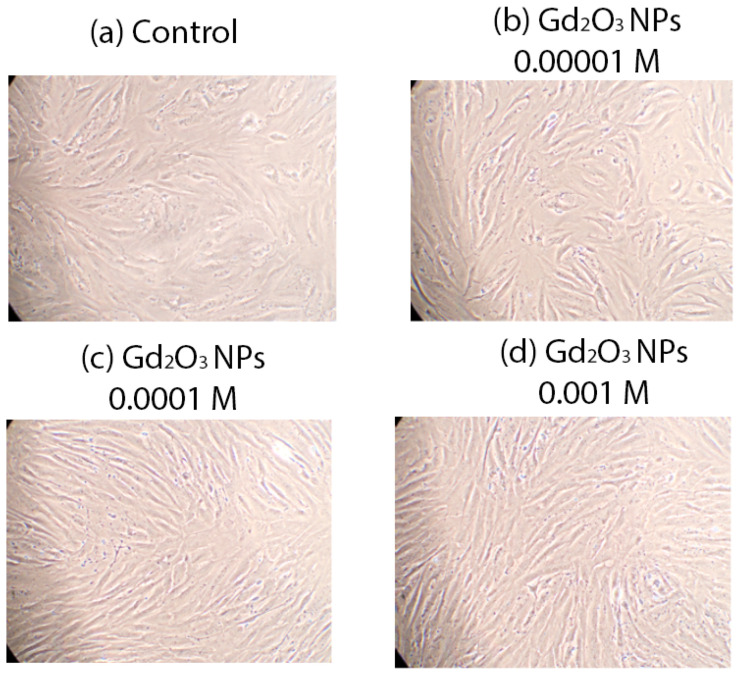
Human fibroblasts after 72 h incubation with different concentrations of Gd_2_O_3_ NPs compared to control, magnification ×20.

Analysis of the percentage of dead cells when Gd_2_O_3_ NPs were co-cultured at different concentrations showed no cytotoxicity. The percentage of dead cells did not exceed 3% and was detected in single (0–1 out of 7, i.e., 0–14%) samples, which indicated the biocompatibility of Gd_2_O_3_ NPs in a wide range of concentrations (Figure 8).

Thus, Gd_2_O_3_ NPs stimulated fibroblast metabolism to the highest degree at concentration of 10^−3^ M (27% enhancement); i.e., it enhanced the production of interstitial substance during regeneration (collagen, elastin) without affecting the proliferation of human fibroblasts, demonstrating high biocompatibility. The complex analysis of the obtained data allows us to give preference to the concentration of 10^−3^ M as the best dose of nanogadolinium for stimulation of fibroblasts for regenerative purposes.

#### 3.3.2. Results of Studies of the Effect of Different Concentrations of Aqueous Suspensions of Gd_2_O_3_ NPs on the Cytotoxicity/Biocompatibility, Metabolic, and Proliferative Activity of Human Keratinocytes

**Figure 9:**
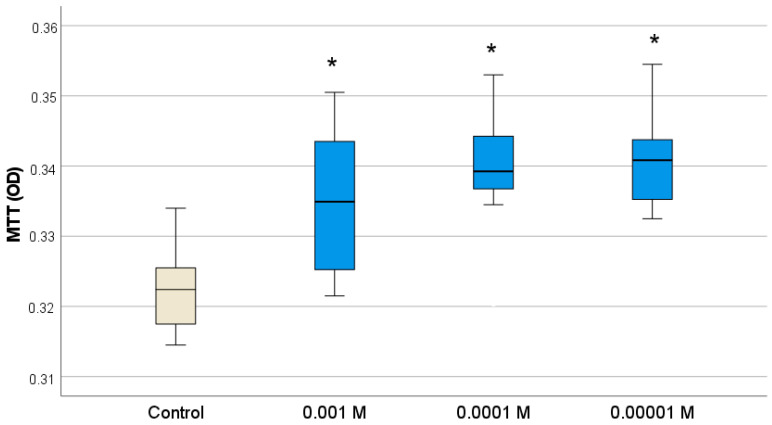
Effect of different concentrations of Gd_2_O_3_ NPs on metabolic activity of human keratinocytes (BJTERT cells) in MTT test (* difference from control at *p* < 0.001, Dunnett post hoc tests).

**Figure 10:**
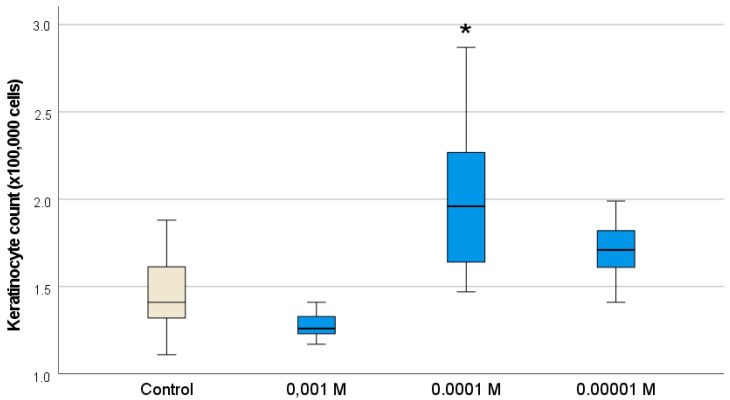
Effect of different concentrations of Gd_2_O_3_ NPs on proliferative activity of fibroblasts (HaCaT cell line) by direct cell counting using an automated cell counter (* difference from control at *p* < 0.001, Dunnett post hoc tests).

It was found that Gd_2_O_3_ NPs also stimulated keratinocyte cells—analogs of the surface layer of the skin. According to the MTT test, Gd_2_O_3_ NPs at all concentrations (10^−3^ M–10^−5^ M) contributed to the intensification of human keratinocyte metabolism (Figure 9), tending to slightly increase activation as the concentration decreased (the highest level of OD was recorded at concentrations of 10^−4^–10^−5^ M). Analysis of the keratinocyte number after 72 h of cultivation proved the growth of proliferative activity of keratinocytes only at one concentration of Gd_2_O_3_ NPs (10^−4^ M). The number of cells was on average 1.37 times higher relative to the control (*p* < 0.01) (Figure 10; Table 3).

**Figure 11:**
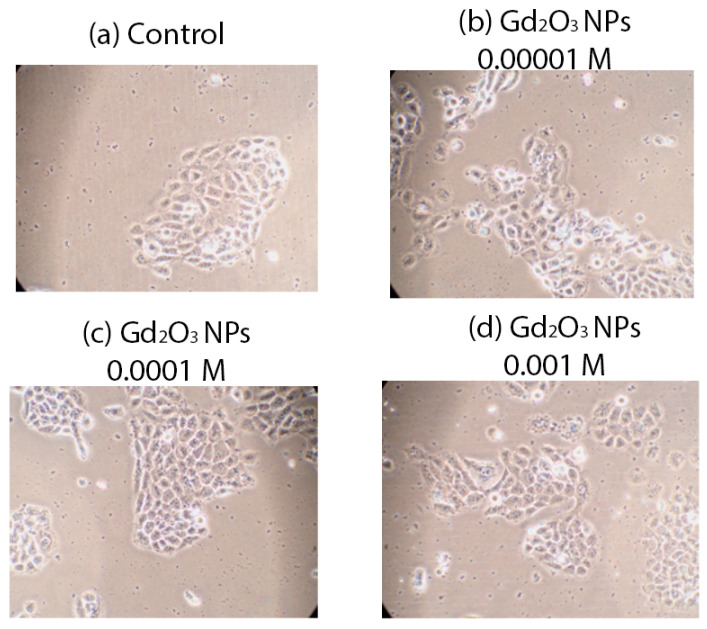
Human keratinocytes after 72 h incubation with different concentrations of Gd_2_O_3_ NPs compared to control, magnification ×20.

A high level of biocompatibility of the investigated concentrations of Gd_2_O_3_ NPs in relation to human keratinocyte culture was determined. There were no dead cells registered in any of the wells. Microscopy of keratinocytes in different groups also did not reveal any negative cytoarchitectonic changes (Figure 11).

Thus, the revealed ability of Gd_2_O_3_ NPs to stimulate human keratinocytes can be the basis for further studies to develop nanomaterials based on Gd_2_O_3_ NPs for skin wound healing. A comprehensive analysis of the tests performed on keratinocytes allows us to choose the best concentration of 10^−4^ M for their stimulation, at which there is activation of both metabolism and proliferative activity of keratinocytes.

#### 3.3.3. Results of the Study of Migration Activity and Healing Rate of a Model Wound In Vitro on Human Mesenchymal Stem Cell Culture

**Figure 12:**
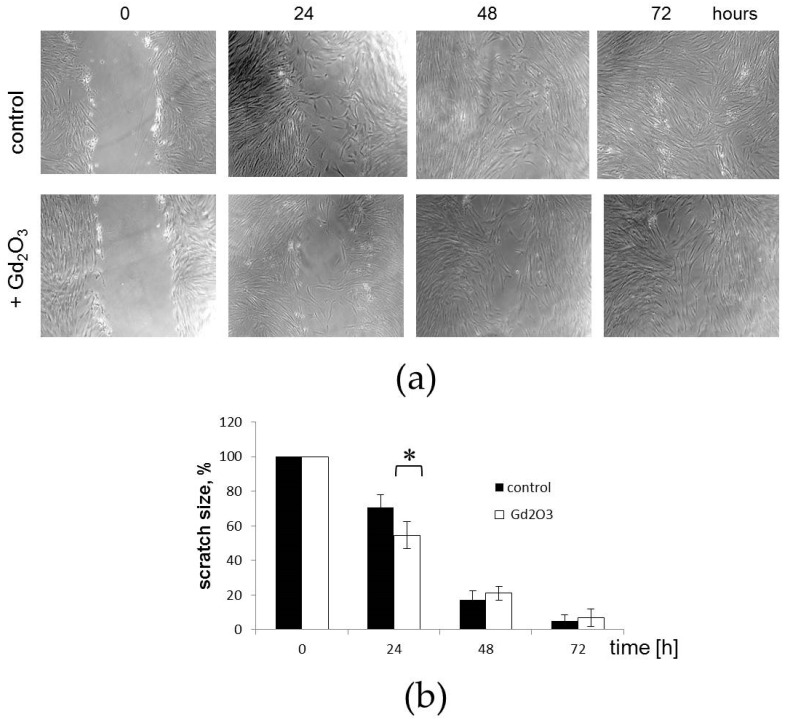
Effects of Gd_2_O_3_ on migration of MSCs in scratch wound healing assay. (**a**) Representative images demonstrate the differences in migratory activity between MSCs under intact conditions and upon the Gd_2_O_3_ treatment. Brightfield microscopy, magnification —40×. (**b**) Time-dependent quantification of % confluency in the scratch wound area (* reliability of differences at *p* < 0.05; *t*-test).

The treatment of MSCs with Gd_2_O_3_ displayed distinct and heterogenic effects on wound scratch closure compared to intact MSCs (Figure 12a). Enhanced migration and more rapid scratch closure were observed in Gd_2_O_3_-treated MSCs compared to their untreated counterparts within 24 h. However, the scratch disappeared by the third day for both treated and resting MSCs (Figure 12b).

Surprisingly, the arrangement of cells in the scratches varied greatly. Individual cells from the untreated MSC line rapidly migrated to the center of the scratch, where they were oriented randomly. At the same time, the cell front at the edges of the scratch remained practically unchanged. In contrast, Gd_2_O_3_-treated MSCs hardly migrated to the center of the scratch. Overgrowth of the scratch in this case occurred due to the collective movement of leading cells. In this case, Gd_2_O_3_-treated MSCs lined up parallel along the scratch (Figure 12a).

These results suggest that Gd_2_O_3_ may not only have a stimulatory effect on scratch healing, but also determine the functional heterogeneity of MSCs.

## 4. Discussion

Nanoscale gadolinium oxide (Gd_2_O_3_) is a promising nanomaterial with unique physicochemical properties that has various applications ranging from biomedicine to catalysis. Most of the current published studies on gadolinium compounds are related to their use as clinical contrast agents for MRI [7,8,9,10,11,12]. However, there are significant safety and toxicity problems associated with the use of nanomaterials in biomedical applications. Assessments of biocompatibility and nanotoxicology are important to support the safe usage of these materials [77,78,79]. Obtaining safer and more efficient nanoparticles for biomedical applications remains a challenge [80,81]. There are published single studies on the physicochemical properties of crystalline complexes of Gd_2_O_3_ NPs without cytotoxic effects on cellular structures [82,83]. Therefore, studies on the effects of nanosized gadolinium oxides on various cell lines over a wide range of concentrations are of significant interest.

In clinical MRI studies, gadolinium-based contrast agent concentrations can range from 10^−2^ M to 10^−5^ M (the average dose 0.08 mL/kg). Many articles use a concentration of gadolinium-based contrast agent in range of 10–100 mM, and this is reflected in the recent reviews [84,85]. In order to evaluate the full range of biological effects, such as cytotoxicity, pro- and antioxidant properties, etc., we used a wide range of particle concentrations from 10^−2^ M to 10^−5^ M. This, in turn, allowed us to find that in the photo-oxidative reaction at a concentration of Gd_2_O_3_ nanoparticles of 10^−2^ M, a qualitative transition of the activity of nanoparticles from antioxidant to pro-oxidant properties is observed.

The conducted study on systematic integrated evaluation of chemical–physical characteristics and biological action of Gd_2_O_3_ NPs provided interesting scientific data that may be useful for further development and potential application of Gd_2_O_3_ NPs nanoparticles not only for tissue visualization for diagnostic purposes, but also for therapeutic medicine.

The gadolinium oxide nanoparticles synthesized by thermal decomposition of gadolinium carbonate precipitated from nitrate solution (this method is suitable for large-scale production) had an average size of 20–30 nm and a relatively high degree of crystallinity with the structure of cubic singony and lattice parameter a = 10.79(9) Å. The spectrum obtained by Raman spectroscopy contained peaks in all three regions described above, and their position agreed well with the position of Raman active modes and those caused by the main vibrations of atoms in the crystal lattice (Gd–Gd and Gd–O) or their combinations, which spoke about the structural quality of Gd_2_O_3_ nanocrystals.

Thus, the analysis performed by instrumental methods confirmed both the chemical composition of the studied substance and its physicochemical characteristics, which made it possible to start studying the effect of gadolinium oxide nanoparticles on biological objects.

It was found that Gd_2_O_3_ NPs were highly biocompatible at a concentration of ≤10^−3^ M, which was attributed to exhibiting antioxidant properties.

However, in the study on cell cultures, we did not obtain a direct correlation of the antioxidant effect to the studied concentration ranges. Nevertheless, increasing the concentration of the studied substance in the growth medium resulted in a significant increase in the proliferative activity of fibroblasts with relatively little metabolic activity. Studies on cell lines showed a high level of safety and regenerative potential of Gd_2_O_3_ NPs, which stimulated fibroblast metabolism at concentrations of 10^−2^ to 10^−3^ M (27–28% enhancement); i.e., they were able to enhance the production of interstitial matter during regeneration (collagen, elastin) without affecting the proliferation of human fibroblasts and demonstrating high biocompatibility. The identified ability of Gd_2_O_3_ NPs to stimulate human keratinocytes may be the basis for further research to develop nanomaterials based on Gd_2_O_3_ NPs for skin wound healing. Complex analysis of the tests performed on keratinocytes allows choosing the best concentration of 10^−4^ M for their stimulation, at which there is activation of both metabolism and proliferative activity of keratinocytes (cell growth is enhanced on average by 37% at the concentration of 10^−4^ M). Lower and higher concentrations of gadolinium nanoparticles (10^−3^ M and 10^−5^ M) did not enhance proliferation growth; on the contrary, at the concentration of 10^−3^ M an unreliable tendency to decrease the growth of keratinocyte cell number was determined.

The obtained data demonstrate the pharmacologic potential of nanogadolinium in wound healing agents. Tissue regeneration is impossible without the participation of multipotent mesenchymal stromal cells (MSCs), as they perform a regulatory function at the site of injury, stimulating cell recruitment, angiogenesis, synthesis, and remodeling of extracellular matrix. In particular, it has been shown that MSCs contribute to the reduction of inflammation and promote successful healing through dynamically changing secretion of cytokines, chemokines, growth factors, extracellular vesicles, and extracellular matrix proteins under the influence of external signals from the microenvironment [86,87]. The obtained data from studies on MSCs with regard to the ability of Gd_2_O_3_ NPs not only to accelerate scratch healing but also to determine the functional heterogeneity of MSCs may bring beneficial effects in regenerative medicine not only for skin wound healing but also for reparative processes of internal organs.

Structural defects, which are detected by the results of studies, being located on the surface of nanoparticles, can perform a functional role as centers on which the uncompensated charge is localized and on which redox reactions can take place. Consequently, the increase of structural defects on the surface can lead to the growth of redox-active centers and increase the chemical activity of nanoparticles.

In our case, increasing the concentration to 10^−2^ M contributed to the pronounced agglomeration of NPs, which did not allow studying the dynamics of cell numbers. This, in turn, contributed to reduce the number of working active centers, due to a decrease in the specific surface area of the agglomerates. This further emphasizes the need for measures to reduce agglomeration of nanoparticles in solutions when developing medical devices. This problem is usually solved by immersing the nanoparticle in a polymer, carbonic acid or polysaccharide to create a thin coating. In our case, when the primary aim was to study the physical parameters of the nanoparticle, we used uncoated nanogadolinium. In addition, the toxic effects of the uncoated particle could be stronger, and the study of this was our second aim.

It is worth noting that, under light irradiation, the reason for the simultaneous pro- and antioxidant activity may have been due to a different mechanism. The nonlinear dependence of the redox effect can be explained by an up-conversion mechanism characteristic of Gd_2_O_3_:Eu^3+^ compounds. Apparently, at high concentrations of Gd_2_O_3_ NPs, the prooxidant effect may prevail over antioxidant processes, but further studies are required to prove and study these mechanisms.

According to Raman spectroscopy and EDX analysis, the structure of Gd_2_O_3_ NPs contains insignificant amounts of impurities that can cause the manifestation of prooxidant properties when the particles are irradiated with red light. However, firstly, the amount of these impurities was small, and we did not find any signs indicating the resulting toxic effects during the studies in cell cultures. Second, the presence of lanthanide impurities can enhance the luminescent properties of Gd_2_O_3_ NPs, which directly depend on the additional impurity energy levels inside the forbidden band Gd_2_O_3_. It is known that Gd_2_O_3_ doped with impurity elements (Eu, Yb, etc.) is a pronounced up-conversion luminophore [88], which is able to excite electron-hole pairs, including luminescent ones, due to multiphoton absorption of low-energy light quanta. In this case, electron-hole pairs can be separated by an energy barrier exceeding the energy of single photons of the excitation radiation. The concentration of impurity elements can be rather low, nevertheless providing a high quantum yield. When Gd_2_O_3_ NPs are irradiated with red light photons, the photogenerated electron-hole pairs with a long lifetime can migrate to the particle surface and initiate a cascade of redox reactions accompanied by the release of active radicals [89]. It appears that at high concentrations of Gd_2_O_3_ NPs, pro-oxidant effects may prevail over antioxidant processes, but further studies are required to prove and explore these mechanisms. This can be and, obviously, will be used in imaging systems in our studies on the distribution and excretion of gadolinium oxide nanoparticles from the animal organism at different methods of its administration (percutaneous, oral, injection, injection into the vascular bed). Conducting lifetime tagging studies will help us answer some of the questions of drug delivery and, hopefully, cancer treatment.

At this stage, our work has shown biocompatibility (absence of cytotoxic effects) of Gd_2_O_3_ NPs in concentrations 10^−3^ M–10^−5^ M on different cell lines involved in wound regeneration: MSCs, fibroblasts, and keratinocytes. The effects useful for regeneration, including in skin wound healing, have been revealed.

The main purpose of this study was to investigate the possibilities of biomedical applications of Gd_2_O_3_ NPs, and we also wanted to answer the question of the toxicity of Gd_2_O_3_ NPs. When planning the work, we set ourselves the task of obtaining nanoparticles with a size of less than 50 nm, and agglomerates up to 100 nm, and the results satisfied us. Whether the size of the final drug affects the effectiveness of therapy remains to be determined in subsequent studies.

## 5. Conclusions

The method of thermal decomposition of gadolinium carbonate used for the preparation of nanosized gadolinium (III) oxide is suitable for large-scale production and results in obtaining high-chemical-purity Gd_2_O_3_ NPs of cubic structure with an average size of 20–30 nm, and the particles have a relatively high degree of crystallinity.

In the study of photooxidative degradation of methylene blue dye in the presence of Gd_2_O_3_ NPs under red light irradiation, it was found that Gd_2_O_3_ nanoparticles showed weakly pronounced antioxidant activity, which depends on the content of particles in the solution. At a concentration of 10^−3^ M, the highest antioxidant activity of Gd_2_O_3_ nanoparticles was observed when the rate constant of the dye photodegradation reaction decreased by 5.5% to 9.4 × 10^−3^ min^−1^. When the concentration of Gd_2_O_3_ NPs in solution was increased to 10^−2^ M upon irradiation with a red light source, their antioxidant activity changed to pro-oxidant activity, accompanied by a 15% increase in the rate of the methylene blue degradation reaction.

Studies on cell lines showed a high level of safety and regenerative potential of Gd_2_O_3_ NPs, which stimulated fibroblast metabolism at a concentration of 10^−3^ M (27% enhancement) without affecting their proliferation. Gd_2_O_3_ NPs stimulated keratinocyte metabolism at concentrations of 10^−3^ M–10^−6^ M, and enhanced keratinocyte proliferation by an average of 35% at concentrations of 10^−4^ M. Gd_2_O_3_ NPs accelerated the migration of MSCs, enhancing their proliferation, and promoting the healing of the model wound.

Thus, the results of this study demonstrated the safety and high regenerative potential of redox-active Gd_2_O_3_ nanoparticles towards different cell lines involved in wound regeneration. This may be the basis for further research to develop nanomaterials based on Gd_2_O_3_ NPs for skin wound healing and in regenerative medicine generally. Comprehensive analysis allowed the selection of 10^−4^ M concentration for the development of a drug for wound healing. The data obtained give hope that gadolinium oxide nanomaterials will expand medical applications in the field of regenerative medicine.

## Figures and Tables

**Table 1:** Mass spectroscopy results in parts per million (ppm).

Element	Gd_2_O_3_	Element	Gd_2_O_3_
Na	<0.5	La	6
Mg	1	Ce	<0.1
Al	5	Pr	0.6
Si	<10	Nd	<0.1
K	0.6	Sm	1
Ca	0.8	Eu	5
Ti	<0.01	Tb	3
V	<0.01	Dy	<0.1
Cr	<0.01	Ho	20
Mn	1	Er	<0.1
Fe	1	Tm	2
Co	<0.02	Yb	<0.1
Ni	<0.02	Pb	<0.2
Cu	1	Bi	<0.2
Zn	2	Th	<0.2
Y	0.6	U	<0.1

**Table 2:** Descriptive statistics of the results of studies on human fibroblast culture when co-cultured with gadolinium oxide nanoparticles at different concentrations.

Group	Mean	Std. Deviation	Std. Error	95% Confidence Interval for Mean		Minimum	Maximum
				Lower	Upper		
	MTT, optical density value (OD)						
Control_0	0.432	0.011	0.005	0.425	0.438	0.423	0.453
Control (H_2_O)	0.441	0.014	0.006	0.433	0.448	0.420	0.462
Control (average)	0.437	0.013	0.004	0.428	0.445	0.420	0.462
Gd_2_O_3_ (10^−3^ M)	0.556	0.012	0.003	0.548	0.563	0.540	0.575
Gd_2_O_3_ (10^−4^ M)	0.479	0.009	0.003	0.473	0.485	0.468	0.493
Gd_2_O_3_ (10^−5^ M)	0.467	0.017	0.005	0.456	0.478	0.435	0.501
	Number of fibroblasts, ×100,000 cells						
Control	4.40	0.453	0.171	3.98	4.82	3.69	4.99
Gd_2_O_3_ (10^−3^ M)	4.71	0.452	0.171	4.29	5.12	4.05	5.34
Gd_2_O_3_ (10^−4^ M)	4.02	0.287	0.108	3.75	4.29	3.76	4.52
Gd_2_O_3_ (10^−5^ M)	4.10	0.930	0.352	3.24	4.96	2.40	5.10

**Table 3:** Descriptive statistics of the results of in vitro studies on human keratinocyte culture when co-cultured with gadolinium oxide nanoparticles at different concentrations.

Group	Mean	Std. Deviation	Std. Error	95% Confidence Interval for Mean		Minimum	Maximum
				Lower	Upper		
	MTT, optical density value (OD)						
Control	0.322	0.007	0.002	0.316	0.329	0.315	0.334
Gd_2_O_3_ (10^−3^ M)	0.335	0.011	0.004	0.324	0.345	0.322	0.351
Gd_2_O_3_ (10^−4^ M)	0.339	0.010	0.003	0.329	0.348	0.321	0.353
Gd_2_O_3_ (10^−5^ M)	0.341	0.007	0.003	0.334	0.348	0.333	0.355
Total	0.334	0.011	0.002	0.330	0.339	0.315	0.355
	Number of keratinocytes, ×100,000 cells						
Control	1.47	0.268	0.101	1.21	1.71	1.11	1.88
Gd_2_O_3_ (10^−3^ M)	1.33	0.157	0.059	1.18	1.47	1.17	1.64
Gd_2_O_3_ (10^−4^ M)	2.02	0.515	0.194	1.53	2.49	1.47	2.87
Gd_2_O_3_ (10^−5^ M)	1.71	0.192	0.072	1.53	1.89	1.41	1.99
Total	1.63	0.399	0.075	1.47	1.78	1.11	2.87

## Data Availability

Details regarding the data supporting the reported results can be found from the first authors.
